# Serum levels of anti-PspA and anti-PspC IgG decrease with age and do not correlate with susceptibility to experimental human pneumococcal colonization

**DOI:** 10.1371/journal.pone.0247056

**Published:** 2021-02-12

**Authors:** Adriano P. Araujo, Gabriela B. C. Colichio, Maria Leonor S. Oliveira, Esther German, Elissavet Nikolaou, Tao Chen, Hugh Adler, Daniela M. Ferreira, Eliane N. Miyaji

**Affiliations:** 1 Laboratório de Bacteriologia, Instituto Butantan, São Paulo, Brazil; 2 Department of Clinical Sciences, Liverpool School of Tropical Medicine, Liverpool, United Kingdom; Public Health England, UNITED KINGDOM

## Abstract

Older adults are at increased risk of pneumococcal disease. This work aims to evaluate whether there is any decrease in serum IgG against variants of the antigens Pneumococcal surface protein A (PspA) and Pneumococcal surface protein C (PspC) in healthy adults with increasing age. Levels of IgG against PspA and PspC variants were determined by ELISA in serum samples comparing volunteers 18–30 years of age with volunteers who were 50–70+ before and after an experimental pneumococcal colonization challenge. The serotype 6B strain used in the challenge belongs to a minor group of pneumococcal isolates expressing two PspC variants. There was a decrease in levels of IgG with increasing age for the most common PspA variants and for all PspC variants analyzed. No correlation was found between basal levels of IgG against these antigens and protection against colonization. There was an increase in levels of IgG against PspA variants that are more cross-reactive with the variant expressed by the challenge strain post challenge in younger individuals who became colonized. Since the challenge strain used in our study expresses two different PspC variants, an increase in serum IgG against all PspC variants tested was observed in younger individuals who became colonized. For some of the antigen variants tested, a decrease in serum IgG was observed in young volunteers who were challenged but did not become colonized. Serum IgG antibodies against PspA and PspC variants thus decrease with age in healthy adults, but there is no correlation between levels of IgG against these antigens and protection against human experimental colonization. Though no correlation between naturally induced serum IgG antibodies against PspA and PspC and protection against colonization was observed, these results do not rule out the protective potential of these antigens as vaccines against pneumococcal infections.

## Introduction

*Streptococcus pneumoniae* is a major human pathogen, causing diseases such as meningitis, sepsis, pneumonia, otitis media and sinusitis. Both the very young and elderly adults are at higher risk of pneumococcal disease. The incidence of community-acquired pneumonia caused by *S*. *pneumoniae* requiring hospitalization was reported to be almost 5 times higher among adults ≥65 years than among younger adults [1]. In older adults, immunosenescence leads to declines in both innate and adaptive immunity, which is thought to contribute to increased susceptibility to pneumococcal disease [2].

Nasopharyngeal colonization is regarded as a necessary step for the onset of disease. Pneumococcal colonization is common in children, but is reported to be rare in older adults [3, 4]. Parenteral immunization of children with pneumococcal conjugate vaccine (PCV) leads to a drastic reduction of nasopharyngeal colonization by vaccine serotypes [5, 6], indicating that vaccine-induced antibodies against capsular polysaccharide (PS) protect against colonization. Natural colonization leads to the development of antibodies against PS and proteins [7, 8], but it is not clear whether these antibodies are responsible for protection against subsequent colonization. Analysis of naturally acquired antibodies has shown that concentrations of IgG against pneumococcal protein virulence factors, as well as against some PS, increase in children due to exposure to pneumococci [9, 10] and then decrease in older healthy adults [11].

Pneumococcal surface protein A (PspA) and Pneumococcal surface protein C (PspC) are important virulence factors that present some variation. High recombination rates have been described within *pspA* and *pspC* loci, indicating that these surface antigens are under selective pressure, being involved in the evasion of the immune system [12, 13] and strongly suggesting a role for protection elicited by these antigens in humans. PspA has an N-terminal variable α-helical domain, followed by a more conserved proline-rich domain, and a C-terminal domain that anchors the protein non-covalently to the surface via interaction with choline residues [14, 15]. PspA has been classified into Family 1 (clades 1 and 2), Family 2 (clades 3, 4 and 5), and Family 3 (clade 6) [16, 17]. More than 90% of strains express PspA from Family 1 or Family 2 [18, 19]. Among the family 2, PspA clade 5 is the least common [20]. PspC has also an N-terminal variable α-helical domain followed by a proline-rich domain, but the C-terminal domain can anchor the protein either through the choline-binding motif or through covalent binding via the LPXTG sortase motif [21]. PspC has been classified into 11 groups: groups 1 to 6 have the choline-binding domain and groups 7 to 11 have the LPXTG motif. One interesting finding is that some strains have two copies of *pspC in tandem*: one with the choline-binding motif and the other with the LPXTG motif [21]. The percentage of strains expressing the different PspC variants has not been extensively evaluated, but one study analyzing 242 colonization strains found that 96.7% of strains possessed a PspC variant with the choline-binding motif and only 1.6% of strains possessed a PspC variant with the LPXTG sortase motif [22].

In this work, we evaluated serum IgG antibodies against the variable α-helical domains of PspA and PspC from different variants before and after a colonization challenge with a 6B strain expressing PspA1, PspC6 and PspC9. These samples provide a unique opportunity to analyze the induction of antibodies against these variable antigens after a known exposure and colonization outcome. We analyzed sera from young and older adults to evaluate whether there was any decrease in antibodies against the different variants of PspA and PspC with increasing age. Our initial hypothesis was that higher levels of serum IgG antibodies specifically against PspA1, PspC6 and/or PspC9 would correlate with protection against colonization.

## Materials and methods

### Ethics statement

Serum samples are from studies performed at the Liverpool School of Tropical Medicine (LSTM, Liverpool, UK). Written informed consent was obtained from all volunteers. Studies were approved by the North West NHS Research Ethics Committee. Samples from young adults are from the control arm of “The effect of live attenuated inactivated influenza vaccine on experimental human pneumococcal carriage study” performed between October 2015 and April 2017 (REC reference 14/NW/1460, Protocol number 4896, EudraCT number 2014-004634-26, IRAS project ID 166674) [23]. Healthy non-smoking volunteers, aged 18–50, were invited to take part. Exclusion criteria were: influenza or pneumococcal vaccination or clinically confirmed disease in the preceding two years; close contact with “high-risk” individuals (children under 5, immunosuppressed, elderly); current febrile illness; use of antibiotics or immune-modulating medication; pregnancy. Baseline characteristics for the subjects of the original study have been published [23, 24]. Characteristics of individuals selected for this study are shown in Table 1. Samples from volunteers aged 50 years and more are from the “Experimental human pneumococcal carriage model (Programme Grant Research): working towards a nasal vaccine for pneumonia. The effect of age on immune function” study performed between June 2016 and October 2018 (REC reference 16/NW/0031, Protocol number 15–053, IRAS project ID 196461) [25]. Healthy adults aged 50–84 years were approached using advertisements, research volunteer databases and primary care patient lists. All participants underwent safety screening including review of primary care records, physical examination, electrocardiography, spirometry, complete blood count and biochemical profile before participation. Participants were excluded if they had regular close contact with children aged <5 years or immunosuppressed adults, an uncontrolled medical comorbidity, recent steroid or antibiotic therapy, a significant smoking history, or a history of culture-proven pneumococcal disease. Baseline characteristics for the subjects of the original study have been published [25]. Characteristics of individuals selected for this study are shown in Table 1. Studies were cosponsored by the Liverpool School of Tropical Medicine and the Royal Liverpool University Hospital, UK. In both studies, pre-challenge samples were collected 5 to 6 days before the experimental nasal inoculation of pneumococcal strain BHN418 (serotype 6B, PspA1, PspC6 and PspC9) [26]. Post challenge samples were collected 27 to 29 days after the challenge. Colonization was evaluated 2, 6, 9, 14, 21 and 29 days after challenge by blood-agar culture from nasal wash samples. The volunteer was considered colonized (colonization positive) if the challenge strain was recovered at any of these time-points. Statistical power to detect age-related immunological differences in volunteers over 50 years was limited by small numbers in each age decile in the original study [25]. This limitation still applies to the current study.

**Table 1:** Characteristics of cohorts.

	Age group				Total
	18–30	50–59	60–69	70+	
**Number of participants included**	30	16	20	14	80
**Number of females**	16 (53.3%)	8 (50.0%)	12 (60.0%)	7 (50.0%)	43 (53.8%)
**Age at inoculation, median (range)**	20.9 (18–30)	55.0 (50–59)	64.9 (61–69)	73.8 (70–80)	48.0 (18–80)

### Recombinant proteins

Recombinant proteins were expressed using pAE 6xHis vectors [27] in *Escherichia coli* BL21(DE3)pLysS. Expressed recombinant PspA and PspC proteins consist of the mature alpha-helical N-terminal domain of PspA from clades 1 to 6 (PspA1α, PspA2α, PspA3α, PspA4α, PspA5α and PspA6α) and PspC from groups 3, 6, 8 and 9 (PspC3α, PspC6α, PspC8α and PspC9α). The clinical isolates of origin of the *pspA* and *pspC* sequences are shown in Table 2 [26, 28–30]. It is worth emphasizing that the recombinant proteins used in this work lack the conserved proline-rich domain of PspA and PspC, in contrast to previous work published by our group evaluating cross-reactivity [28, 30, 31]. Protein expression was induced in mid-log phase cultures by addition of 1 mM IPTG. Recombinant proteins, bearing an N-terminal histidine tag, were purified from the soluble fraction through affinity chromatography using Ni^++^ charged resin (His Trap HP, GE HealthCare) in an Äkta Prime apparatus (GE HealthCare). Elution was carried out with 250 mM imidazole. The purified fractions were analyzed by SDS-PAGE, dialyzed against Tris-HCl 10 mM (pH = 8), NaCl 20 mM, 0.1% glycine and stored at -20°C.

**Table 2:** Strains of origin of *pspA* and *pspC* sequences.

Recombinant protein*	Strain of origin	Country of isolation	Reference	GenBank accession number
PspA1α	435/96	Brazil	[28]	AY082387
PspA2α	371/00	Brazil	[28]	EF649968
PspA3α	259/98	Brazil	[28]	AY082389
PspA4α	255/00	Brazil	[28]	EF649969
PspA5α	122/02	Brazil	[28]	EF649970
PspA6α	347/158	UK	This study	MT188726
PspC3α	491/00	Brazil	[29]	EF424119
PspC6α	BHN418	Sweden	[26]	ASHP00000000.1
PspC8α	HU329/06	Brazil	[30]	GU138630
PspC9α	BHN418	Sweden	[26]	ASHP00000000.1

* Recombinant proteins comprising the mature α-helical domain of PspA clades 1 to 6 and PspC groups 3, 6, 8 and 9.

### Measurement of anti-PspAα and anti-PspCα IgG antibodies by ELISA

ELISA was carried out in high-binding plates coated with 2 μg/mL of recombinant protein. Coating was carried out for 16 hours at 4°C. Plates were then incubated with four dilutions (1:250, 1:500, 1:1000 and 1:2000) of each serum sample for 2 hours at 37°C. Goat anti-human IgG conjugated with horseradish peroxidase (HRP, Sigma) was used as secondary antibody (1:20,000, 1 hour at 37°C). Antibodies were detected with o-phenylenediamine (OPD) and Abs492nm was measured. Arbitrary units (AI) were calculated by multiplying Abs492nm with reciprocal of dilution. Results with Abs492nm <0.1 and >1.0 were discarded. Mean of AI with at least two dilutions of each sample is presented.

### Statistical analysis

ELISA data were log-transformed for statistical analyses. Differences between age groups were analyzed by One-way ANOVA with Tukey’s HSD post hoc test. Differences between colonization positive and colonization negative individuals in the same age group were analyzed by unpaired Student’s *t*-test. Differences between pre- and post-challenge samples were analyzed by paired Student’s *t*-test. Graph Pad Prism 8 was used for analysis with two-sided 0.05 as significance level.

## Results

### Anti-PspAα IgG in serum samples

**Fig 1:**
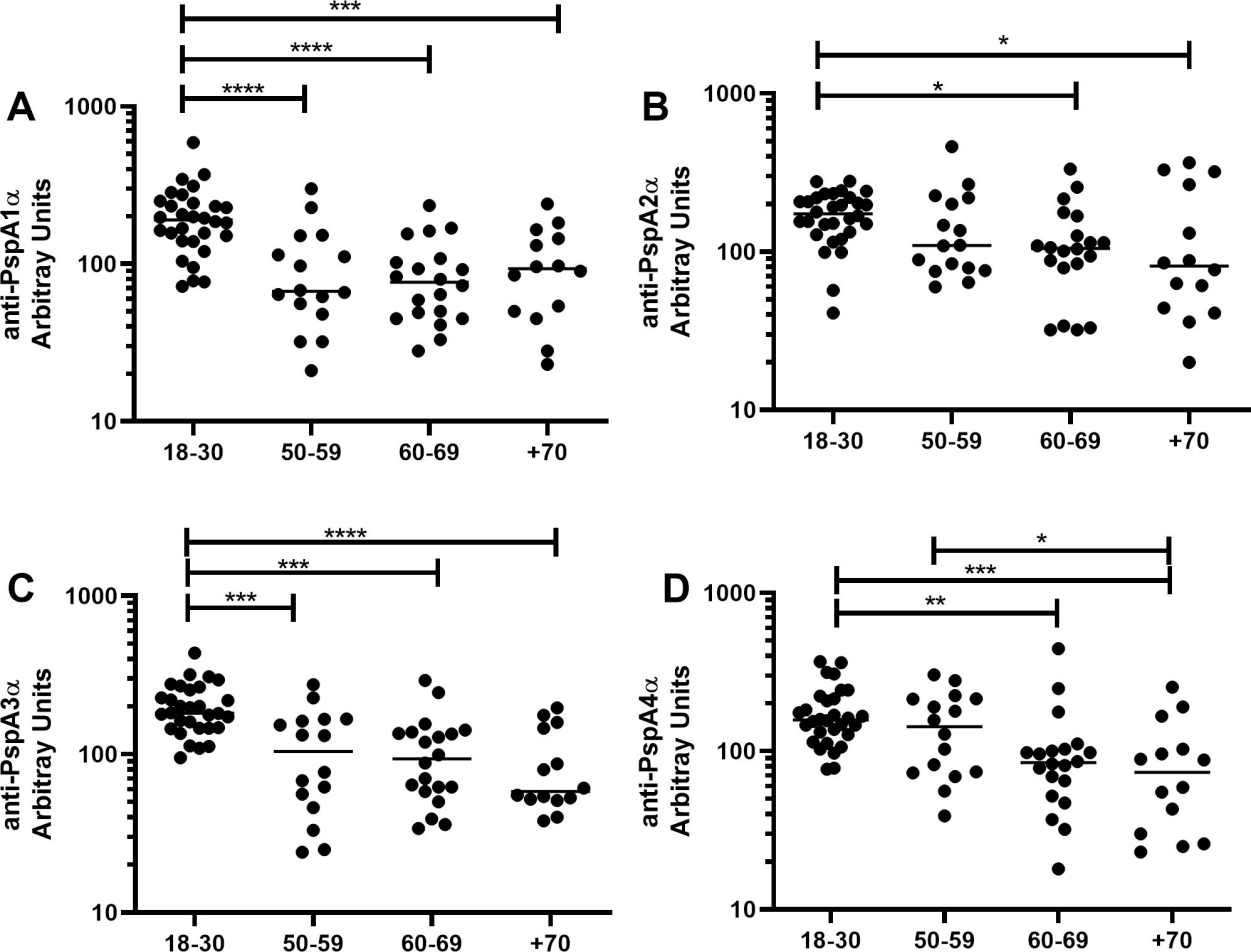
Serum levels of anti-PspA IgG with increasing age. Serum IgG against PspA1α (A), PspA2α (B), PspA3α (C) and PspA4α (D) was detected by ELISA in pre-challenge serum samples of volunteers grouped by age. * indicates difference between age groups with statistical significance (One-way ANOVA with Tukey’s HSD post hoc test, * *P*≤0.05, ** *P*≤0.01, *** *P*≤0.001, **** *P*≤0.0001).

Basal serum levels of IgG anti-PspA1α (Fig 1A), anti-PspA2α (Fig 1B), anti-PspA3α (Fig 1C), anti-PspA4α (Fig 1D), anti-PspA5α (S1A Fig) and anti-PspA6α (S1B Fig) were evaluated in samples from individuals aged 18 to 30 years (18–30 yrs, n = 30), 50 to 59 years (50–59 yrs, n = 16), 60 to 69 years (60–69 yrs, n = 20) and more than 70 years (+70 yrs, n = 14). Higher levels were observed in those 18–30 yrs for anti-PspA1α, anti-PspA2α, anti-PspA3α and anti-PspA4α, which are the common PspA clades expressed by pneumococcal strains. There was no decrease with increasing age for anti-PspA5α and anti-PspA6α serum IgG.

**Fig 2:**
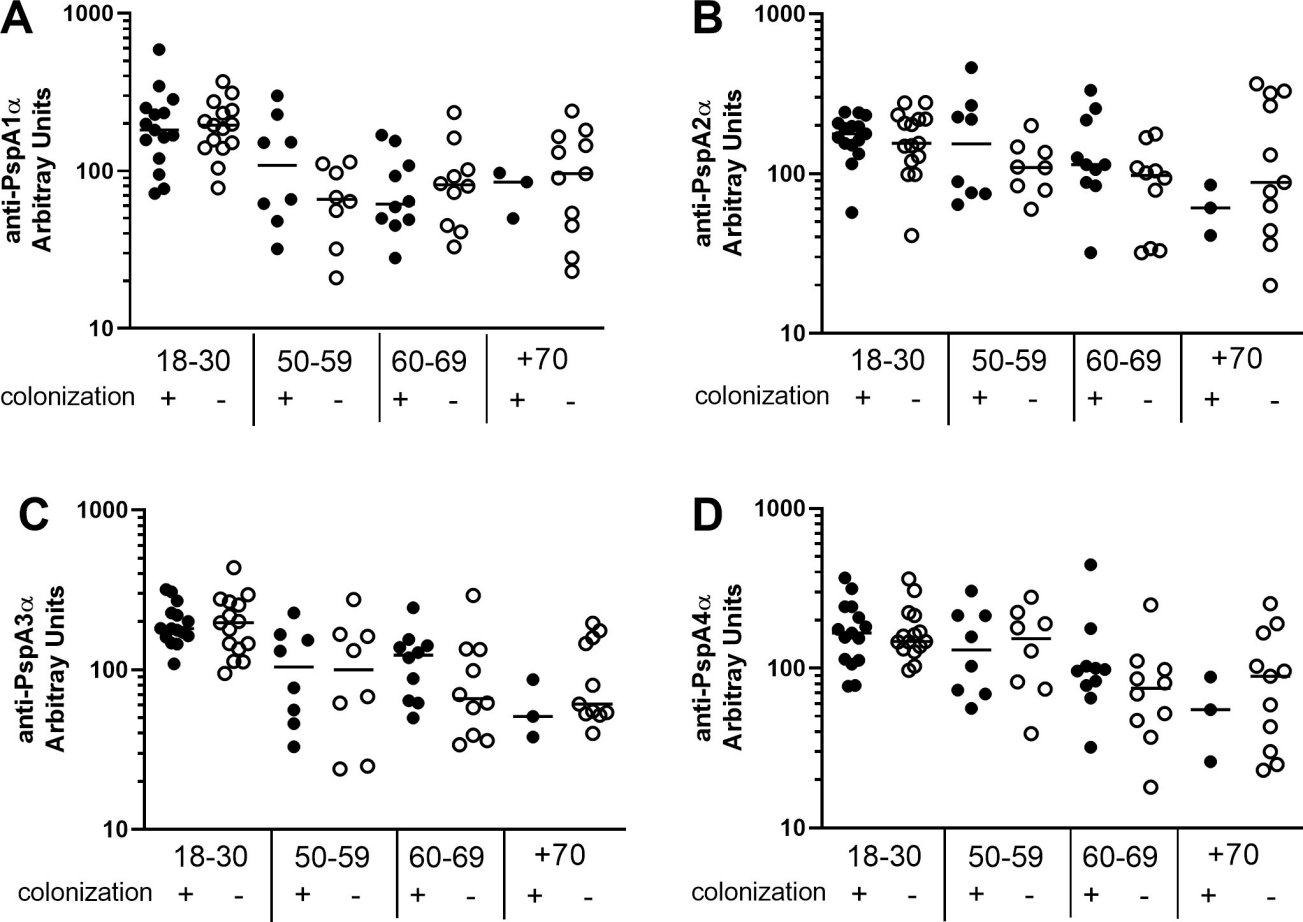
Serum levels of anti-PspA IgG in colonization positive and colonization negative volunteers. Serum IgG against PspA1α (A), PspA2α (B), PspA3α (C) and PspA4α (D) was detected by ELISA in pre-challenge serum samples of colonization positive and colonization negative volunteers grouped by age. Differences between colonization positive and colonization negative volunteers within the same age group were not significant by unpaired Student’s *t*-test.

Differences in basal levels of IgG against the different PspAs between colonization positive and colonization negative volunteers stratified by age were evaluated (18–30 yrs, colonization positive n = 15, colonization negative n = 15; 50–59 yrs, colonization positive n = 8, colonization negative n = 8; 60–69 yrs, colonization positive n = 10, colonization negative n = 10; +70 yrs, colonization positive n = 3, colonization negative n = 11). Levels of antibodies were similar in colonization positive and colonization negative individuals in all age groups, except for higher levels of anti-PspA5α IgG in colonization positive volunteers in the 60–69 yrs group (Fig 2 and S2 Fig).

**Fig 3:**
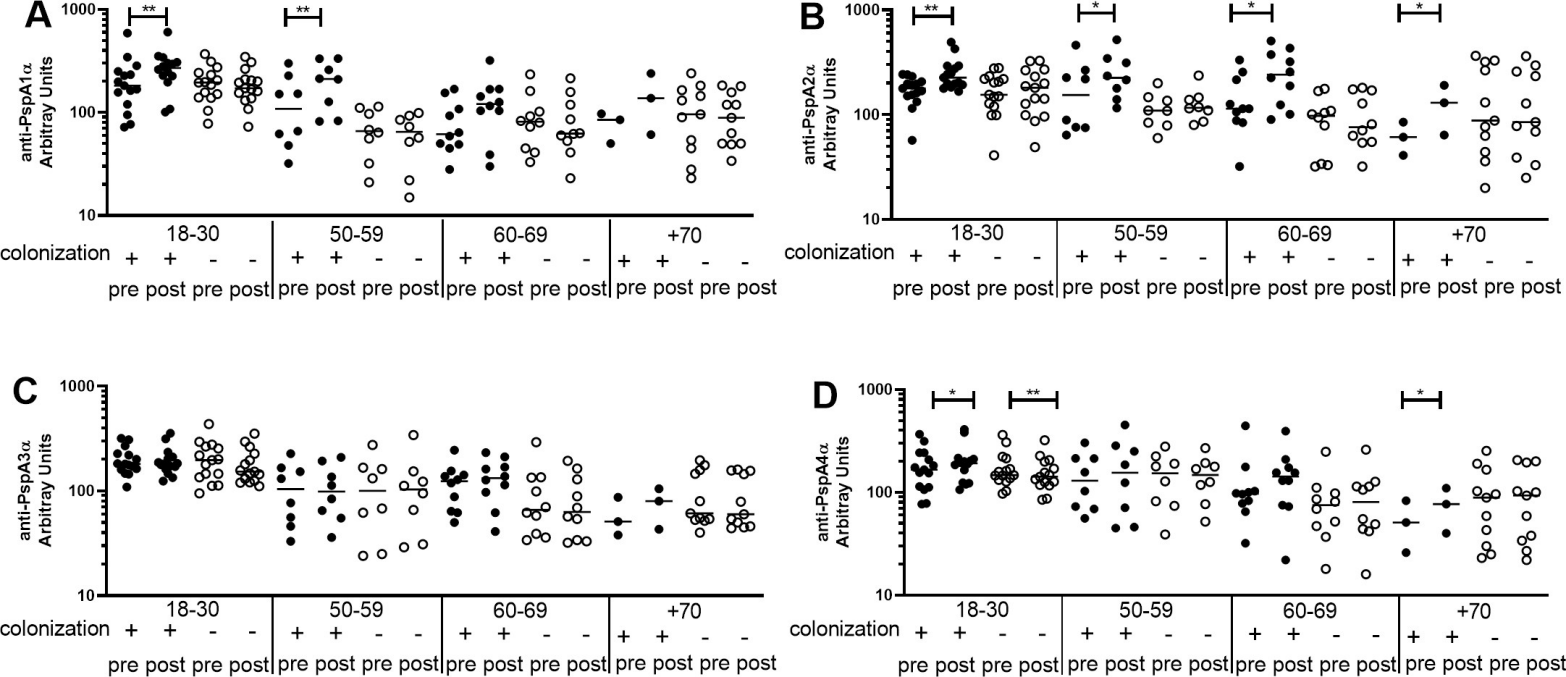
Pre- and post-challenge serum levels of anti-PspA IgG in colonization positive and colonization negative volunteers. Serum IgG against PspA1α (A), PspA2α (B), PspA3α (C) and PspA4α (D) was detected by ELISA in pre- and post-challenge serum samples of colonization positive and colonization negative volunteers grouped by age. * indicates difference with statistical significance between pre- and post-challenge samples (Paired Student’s *t*-test, * *P*≤0.05, ** *P*≤0.01).

Differences between pre- and post-challenge levels of anti-PspA IgG were evaluated by age group in colonization positive and colonization negative individuals. There was an increase in IgG levels only in colonization positive volunteers aged 18–30 yrs and 50–59 yrs for anti-PspA1α, aged 18–30 yrs, 50–59 yrs, 60–69 yrs and +70 yrs for anti-PspA2α and aged 18–30 yrs and +70 yrs for anti-PspA4α (Fig 3A, 3B and 3D). No differences between pre- and post-challenge sera were observed for anti-PspA3α IgG in any age group (Fig 3C). PspA1 and PspA2 belong to Family 1, the same expressed by the challenge strain. Both PspA3 and PspA4 belong to Family 2, but PspA4 is thought to induce antibodies with higher cross-reactivity [31]. Interestingly, anti-PspA4α IgG levels decreased in the youngest group 18–30 yrs in colonization negative individuals (Fig 3D). As for the rarer variants PspA5α and PspA6α, no differences were observed in IgG against PspA5α and there was a decrease in IgG against PspA6α in carriage negative individuals +70 yrs (S3A and S3B Fig). Values for each subject at pre- and postchallenge time points are shown as (S4 Fig).

### Anti-PspCα IgG in serum samples

**Fig 4:**
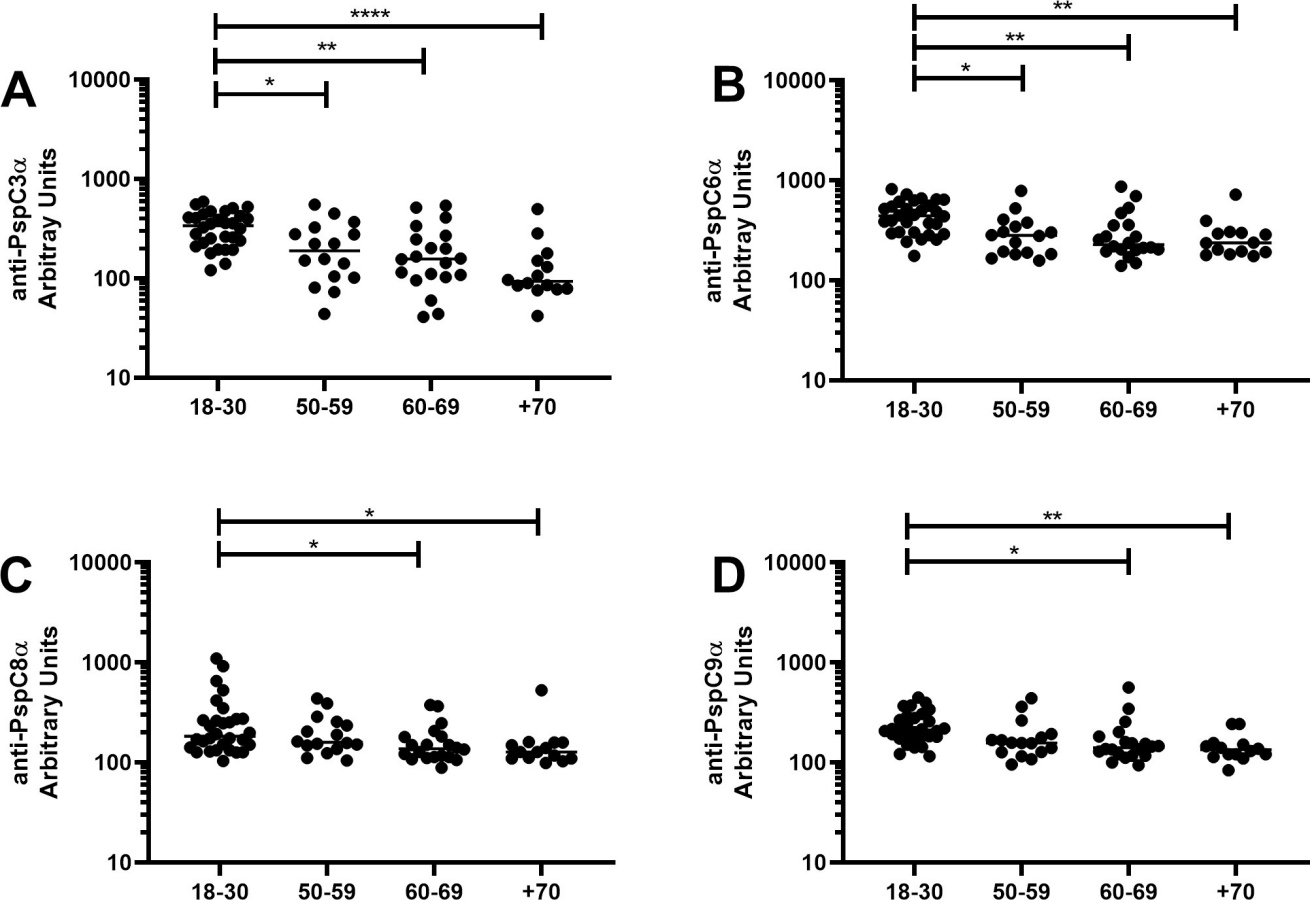
Serum levels of anti-PspC IgG with increasing age. Serum IgG against PspC3α (A), PspC6α (B), PspC8α (C) and PspC9α (D) was detected by ELISA in pre-challenge serum samples of volunteers grouped by age. * indicates difference between age groups with statistical significance (One-way ANOVA with Tukey’s HSD post hoc test, * *P*≤0.05, ** *P*≤0.01, **** *P*≤0.0001).

Basal serum levels of IgG anti-PspC3α (Fig 4A), anti-PspC6α (Fig 4B), anti-PspC8α (Fig 4C) and anti-PspC9α (Fig 4D) were evaluated in samples from individuals aged 18–30 yrs, 50–59 yrs, 60–69 yrs and +70 yrs. Higher levels were observed in those aged 18–30 yrs compared to all older groups for anti-PspC3α and anti-PspC6α. PspC3 and PspC6 are commonly expressed variants with the choline-binding motif for surface attachment. Anti-PspC8α and anti-PspC9α IgG was also higher in those 18–30 yrs compared to those 60–69 yrs and +70 yrs.

**Fig 5:**
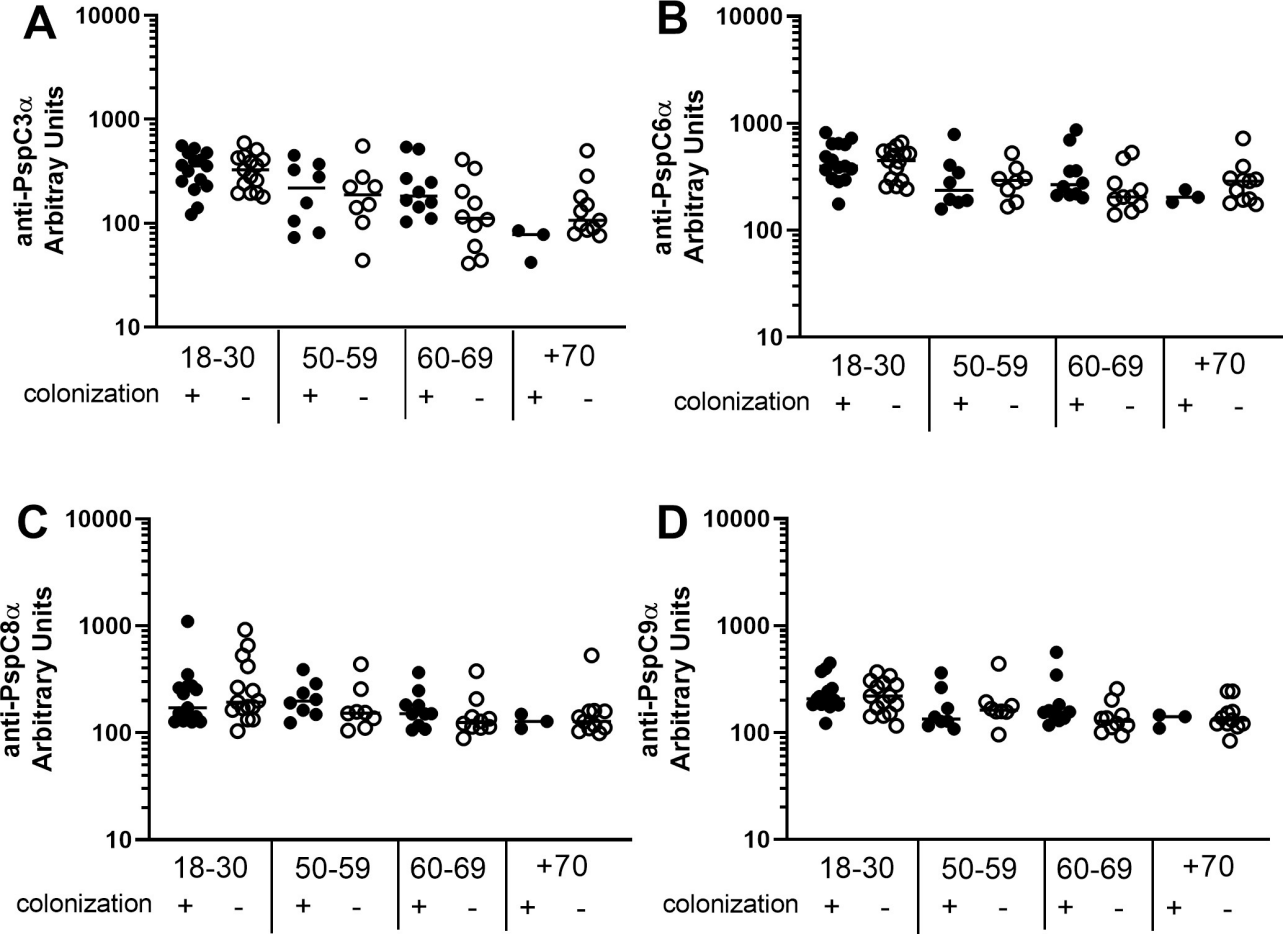
Serum levels of anti-PspC IgG in colonization positive and colonization negative volunteers. Serum IgG against PspC3α (A), PspC6α (B), PspC8α (C) and PspC9α (D) was detected by ELISA in pre-challenge serum samples of colonization positive and colonization negative volunteers grouped by age. Differences between colonization positive and colonization negative volunteers within the same age group were not significant by unpaired Student’s *t*-test.

Differences in basal levels of IgG against the different PspCs between colonization positive and colonization negative volunteers stratified by age were evaluated. Antibody levels were similar in colonization positive and colonization negative individuals in all age groups (Fig 5).

**Fig 6:**
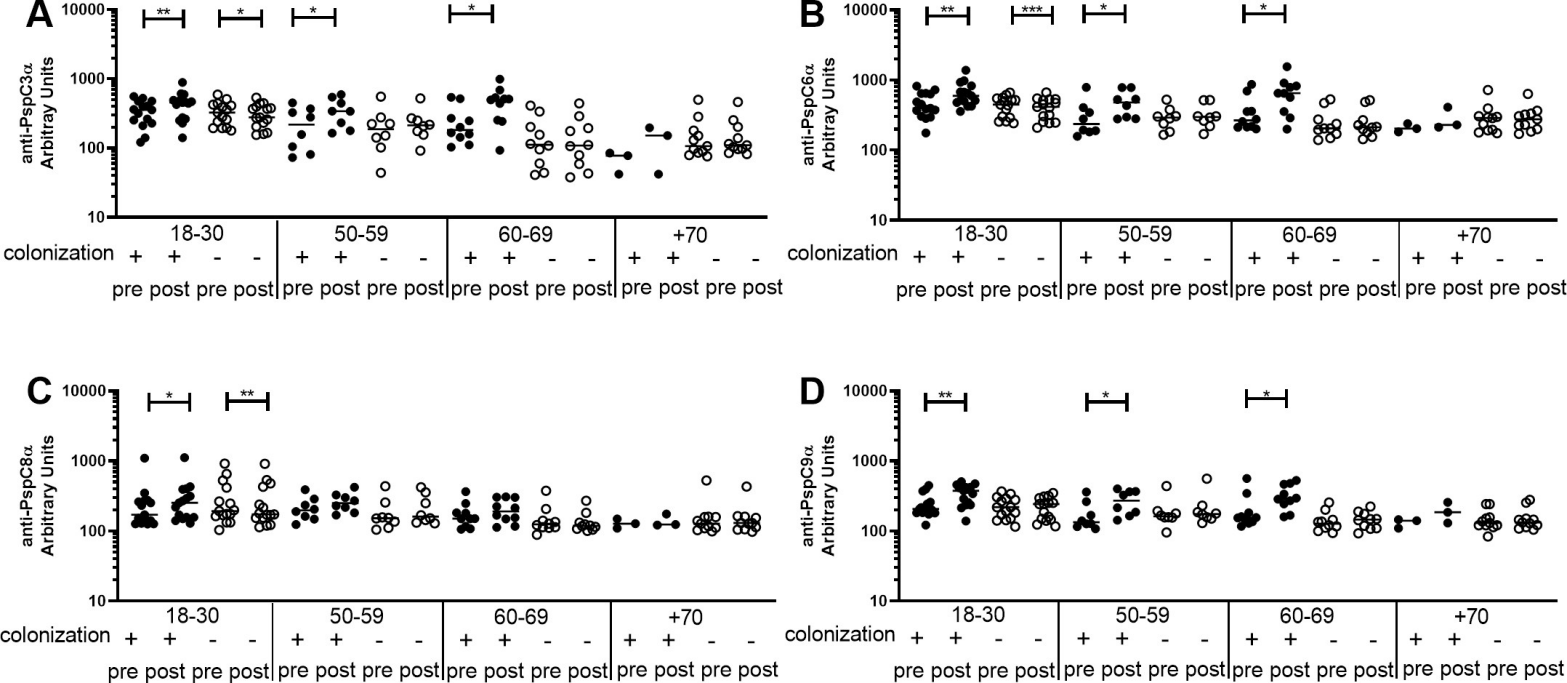
Pre- and post-challenge serum levels of anti-PspC IgG in colonization positive and colonization negative volunteers. Serum IgG against PspC3α (A), PspC6α (B), PspC8α (C) and PspC9α (D) was detected by ELISA in pre- and post-challenge serum samples of colonization positive and colonization negative volunteers grouped by age. * indicates difference with statistical significance between pre- and post-challenge samples (Paired Student’s *t*-test, * *P*≤0.05, ** *P*≤0.01, *** *P*≤0.001).

Differences between pre- and post-challenge levels of anti-PspC IgG were evaluated by age group in colonization positive and colonization negative individuals. There was an increase only in colonization positive volunteers aged 18–30 yrs, 50–59 yrs and 60–69 yrs for anti-PspC3α (Fig 6A), anti-PspC6α (Fig 6B) and anti-PspC9α (Fig 6D), and 18–30 yrs for anti-PspC8α (Fig 6C). There was a decrease in levels of anti-PspC IgG for colonization negative individuals in the youngest group for anti-PspC3α, anti-PspC6α and anti-PspC8α. Values for each subject at pre- and postchallenge time points are shown as (S5 Fig).

## Discussion

Since older adults are at increased risk of pneumococcal disease, we sought to analyze whether there is a decrease in serum levels of IgG against different variants of PspA and PspC in healthy adults with increasing age and also whether there is correlation between serum levels of IgG against these antigens and protection against colonization. We analyzed serum levels of IgG from younger adults and compared to adults with more than 50 years. We had already shown using experimental pneumococcal colonization, that the results for older adults had similar rates, densities, and durations of colonization as in younger adults. Though colonization rates in the oldest participants (ages 70–80 years) were non-significantly lower than those seen in under-50s, low numbers precluded definitive conclusions [25].

We observed decrease in baseline serum levels of IgG against PspA1α, PspA2α, PspA3α and PspA4α with increasing age. No decrease was found for PspA5α and PspA6α. PspA5α and PspA6α are rarer variants of the antigen. A decrease was also observed in serum levels of IgG against PspC3α, PspC6α, PspC8α and PspC9α with increasing age. Previous work has shown that naturally acquired IgG against pneumococcal protein virulence factors, as well as against some PS, decrease with increasing age in healthy adults, including PspA from Family 1, PspA from Family 2 and PspC3 [11]. More recent work confirmed that older individuals have lower serum levels of IgG against PspC3 [32]. Our work is thus in agreement with published data and furthermore shows that smaller differences are observed for rarer variants, probably due to lower exposure to these antigens in younger individuals. Decrease in antibody levels with increasing age in healthy adults may be related both to weaker responses to pneumococcal antigens and to lower exposure to pneumococci.

Our results did not show correlation between serum levels of IgG against any of the PspA and PspC variants and protection against colonization, not even for antibodies against the antigens expressed by the challenge strain, PspA1, PspC6 and PspC9. Previous work with young individuals challenged with the same 6B strain has indeed shown that there is no correlation between pre-challenge serum levels of anti-PS or anti-protein IgG and protection against colonization [33]. Analysis of older adults also failed to find any correlation between anti-PS or anti-protein IgG and protection against colonization [25]. Rather, protection from colonization in young adults in this model was associated with a high number of circulating 6B PS IgG–secreting memory B cells at baseline [34]. It is possible that local T-cell immunity or even local antibody production in the nasal tissue may be more important against pneumococcal colonization than serum antibody levels. Moreover, protection may be correlated with higher overall titers of antibodies against the challenge strain.

One early study using an experimental challenge found that 7 of 8 of the individuals who did not become colonized had serum antibodies against PspA [35]. In the study, the 23F strain used in the challenge had a truncated PspA, which had lost the choline-binding domain of the protein and was not surface-bound, which may account for the differences with our work. Another study evaluating antibody levels against 28 proteins in 242 serum samples found a correlation between lower levels of antibody against PspC variant I (PspC2, 3 and 6) and colonization with a strain expressing this same variant [22]. A larger cohort was analyzed in the study, which may explain the differences with our work. In fact, the authors discuss that despite using a large sample of pneumococcal strains and sera from those who carried these strains, only modest evidence for variant specificity of protection against pneumococcal colonization was found. Furthermore, the fact that the 6B strain used in our work expresses both PspC6 and PspC9 may help bacteria evade the host humoral response and produce confounding results. In fact, successful lineages in colonization and disease were shown to express two PspC variants [26, 36].

We also analyzed serum levels of IgG against the PspA and PspC variants after challenge. Earlier work using this model has shown that antibodies against protein were boosted both in colonization positive and colonization negative individuals. The increase was more pronounced in colonization positive individuals, with statistical difference for PspA UAB055 (PspA2, Family 1) and PspC3, but not PspA UAB099 (PspA3, Family 2) [33]. Titers of antibodies against several proteins were also shown to increase in colonized older adults, including PspA UAB055 (PspA2, Family 1) and PspC3 [25]. Here, we observed an increase in anti-PspA serum IgG only in colonization positive individuals. There was a clear rise in the younger groups post colonization, especially against PspA1α and PspA2α, which are Family 1, the same expressed by the challenge strain. There was also an increase in the 18–30 yrs group for anti-PspA4α, which belongs to Family 2, but has been shown to cross-react with both Family 1 and Family 2 PspA [28, 31]. Interestingly, there was a decrease in levels of IgG anti-PspA4α (Family 2) after challenge in colonization negative individuals 18–30 yrs. We hypothesize that anti-PspA antibodies are sequestered by the challenge strain, leading to decreased levels shortly after contact with bacteria. Colonization with the strain expressing PspA1 then leads to a strong boost to more cross-reactive antigens (PspA1α, PspA2α and PspA4α). On the other hand, those that did not become colonized have either a weak boost with sustained levels of IgG for some variants or no booster at all with decrease in levels of IgG for other variants after longer periods. When analyzing anti-PspC antibodies, there was an increase in levels of IgG against all variants (PspC3α, PspC6α, PspC8α and PspC9α) in younger individuals who became colonized. The increase in antibodies against the four variants can be explained by a booster by the challenge strain expressing both PspC6 and PspC9. There was again a decrease in antibody levels in colonization negative individuals 18–30 yrs, observed now for PspC3α, PspC6α and PspC8α.

One of the limitations of this study is the number of individuals analyzed, especially in the group of individuals +70 yrs who became colonized. Still, we were able to detect a decrease in antibodies against the more common variants of PspA and all PspC variants with increasing age. Furthermore, an increase in antibody levels was detected in younger individuals who became colonized. The increase was observed for PspA variants that are more cross-reactive with PspA1, expressed by the challenge strain, and against all PspC variants tested, which can be explained by the expression of two PspC variants by the challenge strain. Unexpectedly, there was a decrease in serum levels of IgG against some variants in young individuals that did not become colonized. It would be interesting to evaluate whether this decrease has implications in the susceptibility against pneumococcal infections after several episodes of exposure to pneumococci expressing different PspA and PspC variants. No correlation was observed between serum levels of IgG against these antigens and protection against an experimental colonization challenge with pneumococci. These results are similar to what is observed for anti-PS antibodies, with no correlation between naturally induced serum IgG antibodies against PS and protection against experimental colonization, even though immunization with conjugate vaccines clearly induce anti-PS antibodies that provide protection against invasive disease and colonization by vaccine-type pneumococci. Thus, the fact that no correlation was observed between serum levels of IgG against different PspA and PspC variants and protection against colonization does not rule out that vaccination with these antigens may be protective, as observed in animal models.

## Supporting information

S1 Fig Serum levels of anti-PspA IgG with increasing age. Serum IgG against PspA5α (A) and PspA6α (B) was detected by ELISA in pre-challenge serum samples of volunteers grouped by age. Differences between groups were not significant by One-way ANOVA.
(PDF)

S2 Fig Serum levels of anti-PspA IgG in colonization positive and colonization negative volunteers. Serum IgG against PspA5α (A) and PspA6α (B) was detected by ELISA in pre-challenge serum samples of colonization positive and colonization negative volunteers grouped by age. * indicates difference with statistical significance between colonization positive and colonization negative volunteers within the same age group (Unpaired Student’s *t*-test, ** *P*≤0.01).
(PDF)

S3 Fig Pre- and post-challenge serum levels of anti-PspA IgG in colonization positive and colonization negative volunteers. Serum IgG against PspA5α (A) and PspA6α (B) was detected by ELISA in pre- and post-challenge serum samples of colonization positive and colonization negative volunteers grouped by age. * indicates difference with statistical significance between pre- and post-challenge samples (Paired Student’s *t*-test, * *P*≤0.05).
(PDF)

S4 Fig Pre- and post-challenge serum levels of anti-PspA IgG in each colonization positive and colonization negative volunteer. Serum IgG against PspA1α (A), PspA2α (B), PspA3α (C), PspA4α (D), PspA5α (E) and PspA6α (F) was detected by ELISA in pre- and post-challenge serum samples of each colonization positive (+) and colonization negative (-) volunteer grouped by age. * indicates difference with statistical significance between pre- and post-challenge samples (Paired Student’s *t*-test, * *P*≤0.05, ** *P*≤0.01).
(PDF)

S5 Fig Pre- and post-challenge serum levels of anti-PspC IgG in each colonization positive and colonization negative volunteer. Serum IgG against PspC3α (A), PspC6α (B), PspC8α (C) and PspC9α (D) was detected by ELISA in pre- and post-challenge serum samples of each colonization positive (+) and colonization negative (-) volunteer grouped by age. * indicates difference with statistical significance between pre- and post-challenge samples (Paired Student’s *t*-test, * *P*≤0.05, ** *P*≤0.01, *** *P*≤0.001).
(PDF)
