# Supplementary material for: Serum levels of anti-PspA and anti-PspC IgG decrease with age and do not correlate with susceptibility to experimental human pneumococcal colonization
Source: PLoS One. 2021 Feb 12;16(2):e0247056. doi: 10.1371/journal.pone.0247056 (PMC7880446; doi:10.1371/journal.pone.0247056)
Supplement: S1 Fig — Serum IgG against PspA5α (A) and PspA6α (B) was detected by ELISA in pre-challenge serum samples of volunteers grouped by age. Differences between groups were not significant by One-way ANOVA. (PDF) [file pone.0247056.s001.pdf]

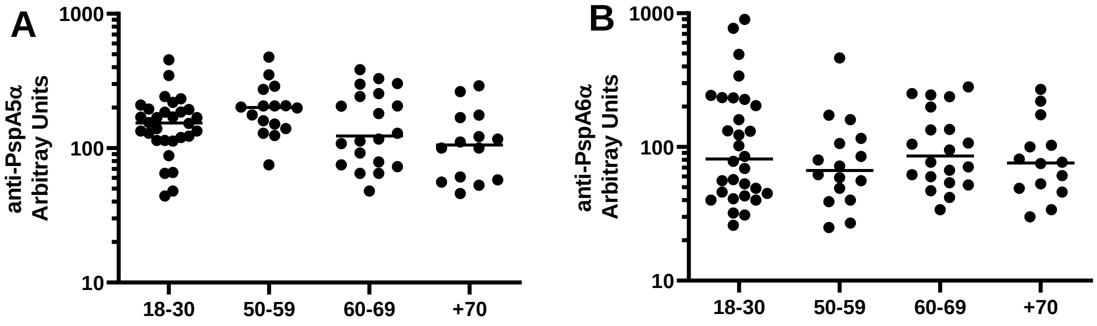

S1 Fig. Serum levels of anti-PspA IgG with increasing age. Serum IgG against PspA5α (A) and PspA6α (B) was detected by ELISA in pre-challenge serum samples of volunteers grouped by age. Differences between groups were not significant by One-way ANOVA.
