# Supplementary material for: Serum levels of anti-PspA and anti-PspC IgG decrease with age and do not correlate with susceptibility to experimental human pneumococcal colonization
Source: PLoS One. 2021 Feb 12;16(2):e0247056. doi: 10.1371/journal.pone.0247056 (PMC7880446; doi:10.1371/journal.pone.0247056)
Supplement: S2 Fig — Serum IgG against PspA5α (A) and PspA6α (B) was detected by ELISA in pre-challenge serum samples of colonization positive and colonization negative volunteers grouped by age. * indicates difference with statistical significance between colonization positive and colonization negative volunteers within the same age group (Unpaired Student’s t-test, ** P≤0.01). (PDF) [file pone.0247056.s002.pdf]

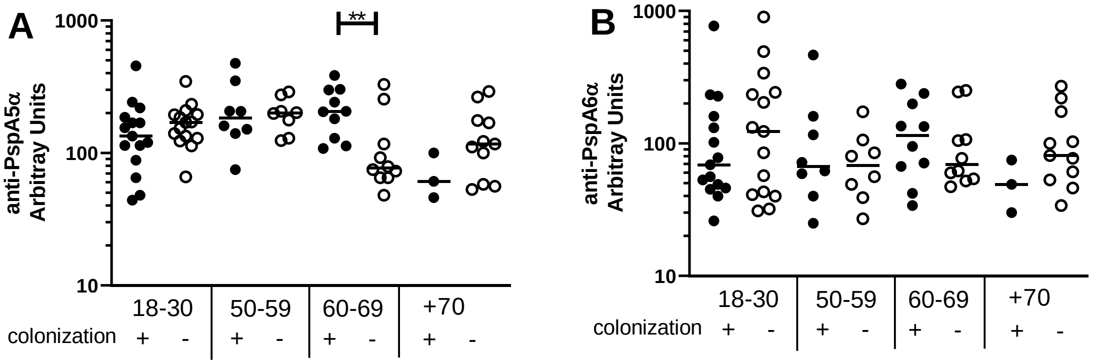

S2 Fig. Serum levels of anti-PspA IgG in colonization positive and colonization negative volunteers. Serum IgG against PspA5α (A) and PspA6α (B) was detected by ELISA in pre-challenge serum samples of colonization positive and colonization negative volunteers grouped by age. * indicates difference with statistical significance between colonization positive and colonization negative volunteers within the same age group (Unpaired Student's *t*-test, ** $P \leq 0.01$).
