# Supplementary material for: Serum levels of anti-PspA and anti-PspC IgG decrease with age and do not correlate with susceptibility to experimental human pneumococcal colonization
Source: PLoS One. 2021 Feb 12;16(2):e0247056. doi: 10.1371/journal.pone.0247056 (PMC7880446; doi:10.1371/journal.pone.0247056)
Supplement: S3 Fig — Serum IgG against PspA5α (A) and PspA6α (B) was detected by ELISA in pre- and post-challenge serum samples of colonization positive and colonization negative volunteers grouped by age. * indicates difference with statistical significance between pre- and post-challenge samples (Paired Student’s t-test, * P≤0.05). (PDF) [file pone.0247056.s003.pdf]

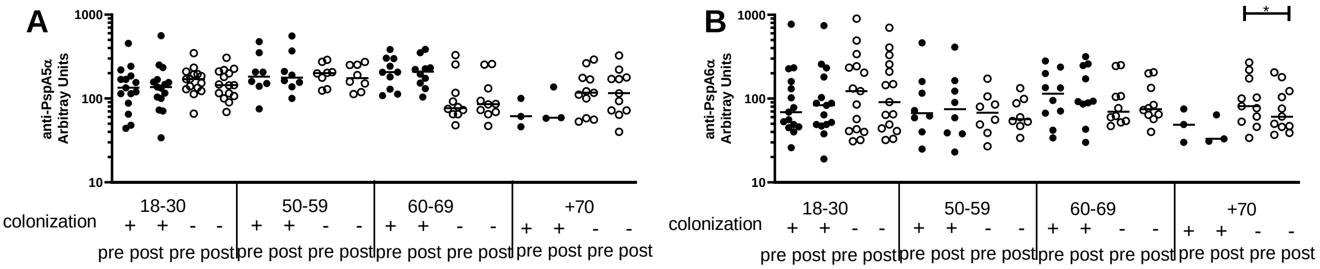


**S3 Fig. Pre- and post-challenge serum levels of anti-PspA IgG in colonization positive and colonization negative volunteers.** Serum IgG against PspA5α (A) and PspA6α (B) was detected by ELISA in pre- and post-challenge serum samples of colonization positive and colonization negative volunteers grouped by age. * indicates difference with statistical significance between pre- and post-challenge samples (Paired Student's *t*-test, * $P \leq 0.05$).
