# Supplementary material for: Serum levels of anti-PspA and anti-PspC IgG decrease with age and do not correlate with susceptibility to experimental human pneumococcal colonization
Source: PLoS One. 2021 Feb 12;16(2):e0247056. doi: 10.1371/journal.pone.0247056 (PMC7880446; doi:10.1371/journal.pone.0247056)
Supplement: S4 Fig — Serum IgG against PspA1α (A), PspA2α (B), PspA3α (C), PspA4α (D), PspA5α (E) and PspA6α (F) was detected by ELISA in pre- and post-challenge serum samples of each colonization positive (+) and colonization negative (-) volunteer grouped by age. * indicates difference with statistical significance between pre- and post-challenge samples (Paired Student’s t-test, * P≤0.05, ** P≤0.01). (PDF) [file pone.0247056.s004.pdf]

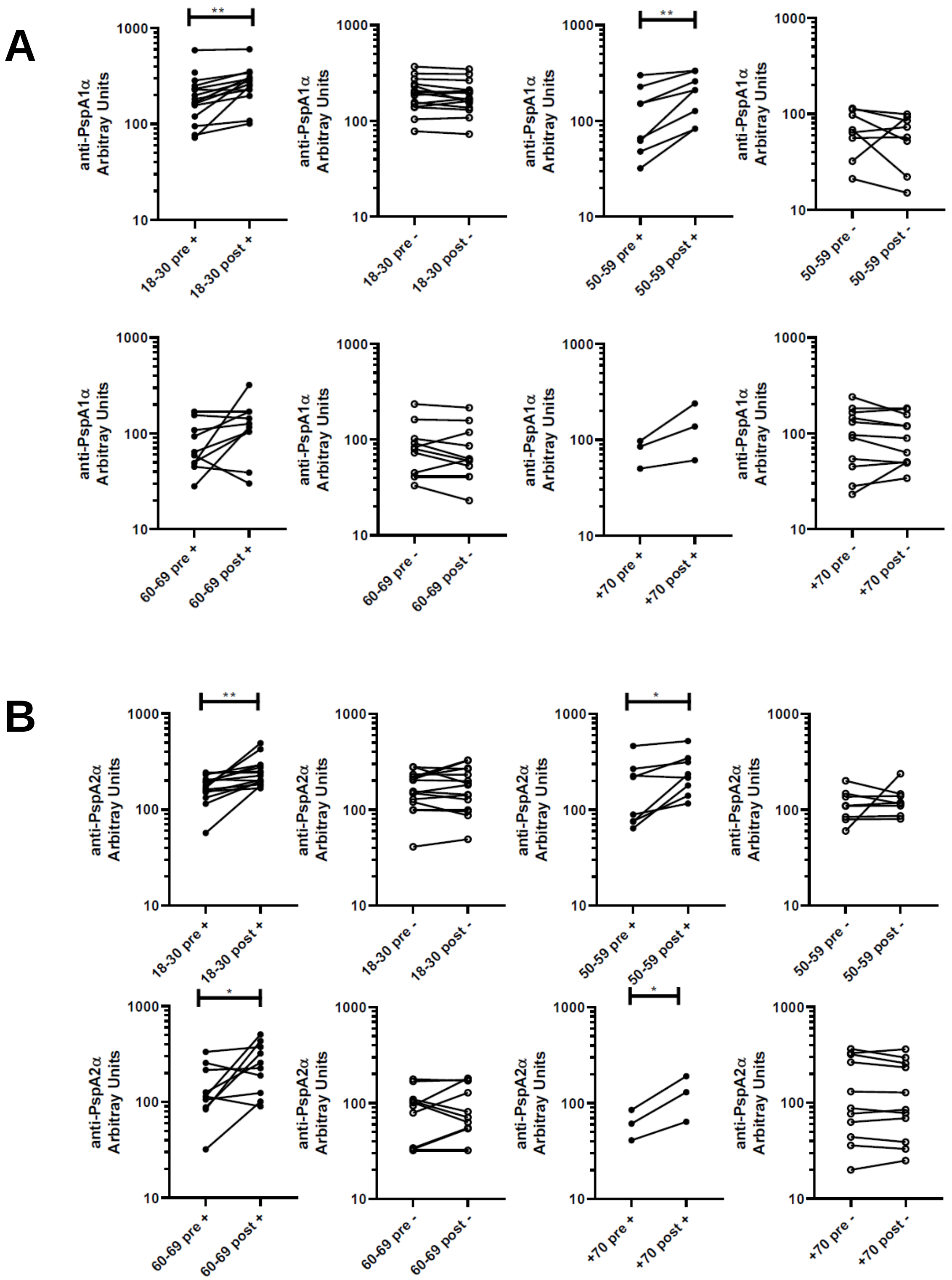

A
**
anti-PspA1α Arbitray Units
1000
100
10
18-30 pre +
18-30 post +
18-30 pre -
18-30 post -
50-59 pre +
50-59 post +
50-59 pre -
50-59 post -
60-69 pre +
60-69 post +
60-69 pre -
60-69 post -
+70 pre +
+70 post +
+70 pre -
+70 post -
B
*
anti-PspA2α Arbitray Units

C

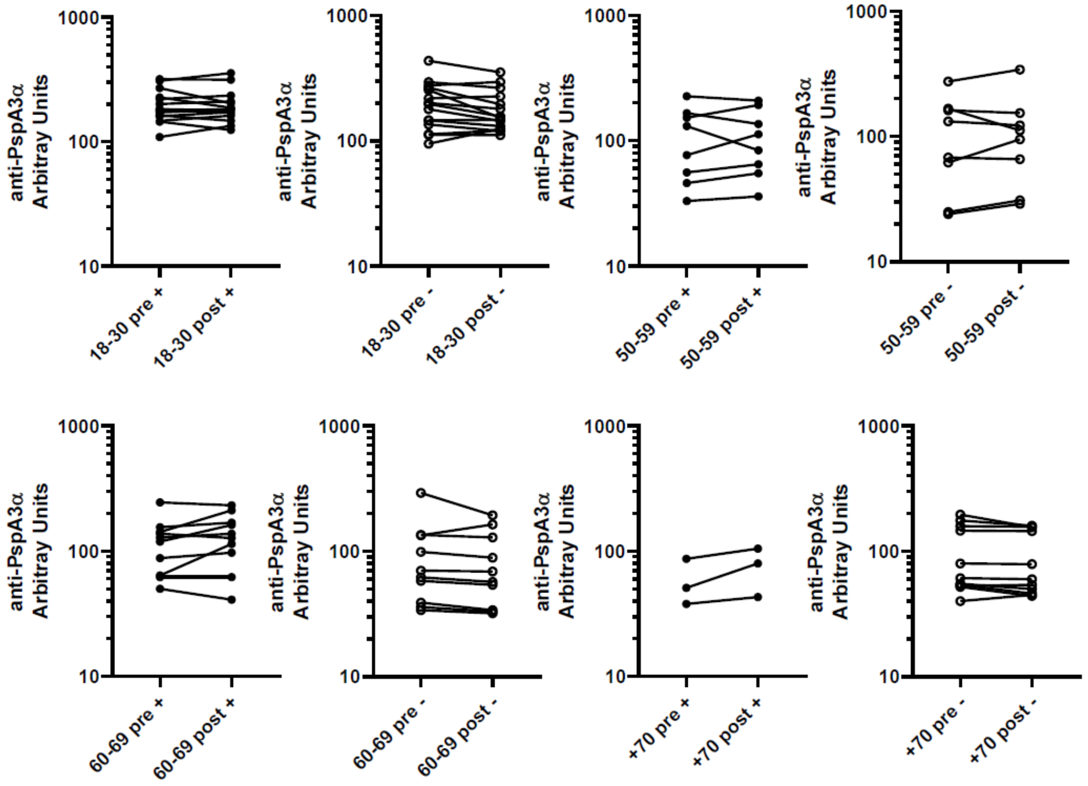
anti-PspA3α Arbitray Units
1000
100
10
18-30 pre +
18-30 post +
18-30 pre -
18-30 post -
50-59 pre +
50-59 post +
50-59 pre -
50-59 post -
60-69 pre +
60-69 post +
60-69 pre -
60-69 post -
+70 pre +
+70 post +
+70 pre -
+70 post -

D

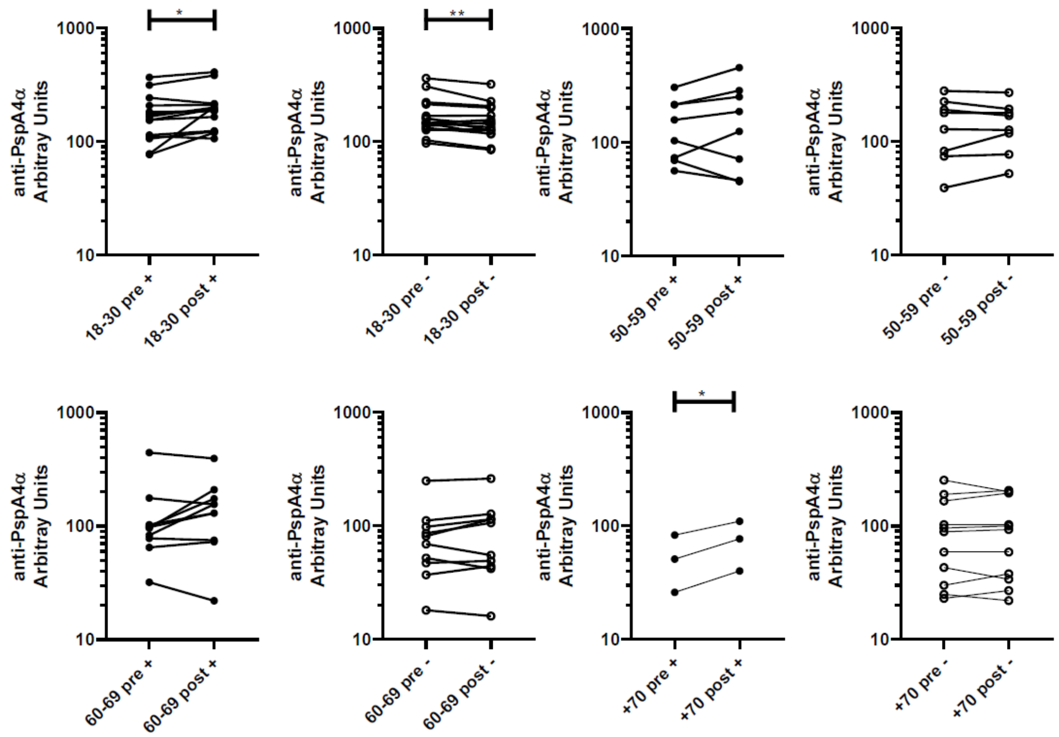
anti-PspA4α Arbitray Units
1000
100
10
*
**
18-30 pre +
18-30 post +
18-30 pre -
18-30 post -
50-59 pre +
50-59 post +
50-59 pre -
50-59 post -
60-69 pre +
60-69 post +
60-69 pre -
60-69 post -
+70 pre +
+70 post +
+70 pre -
+70 post -

E

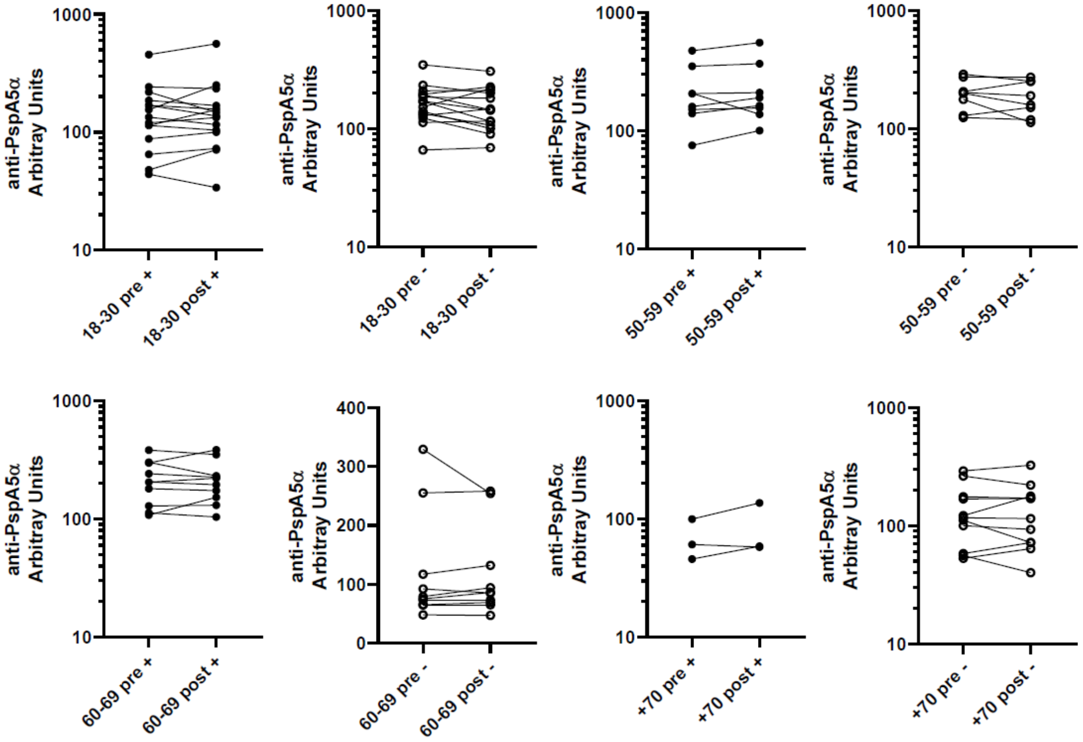

F

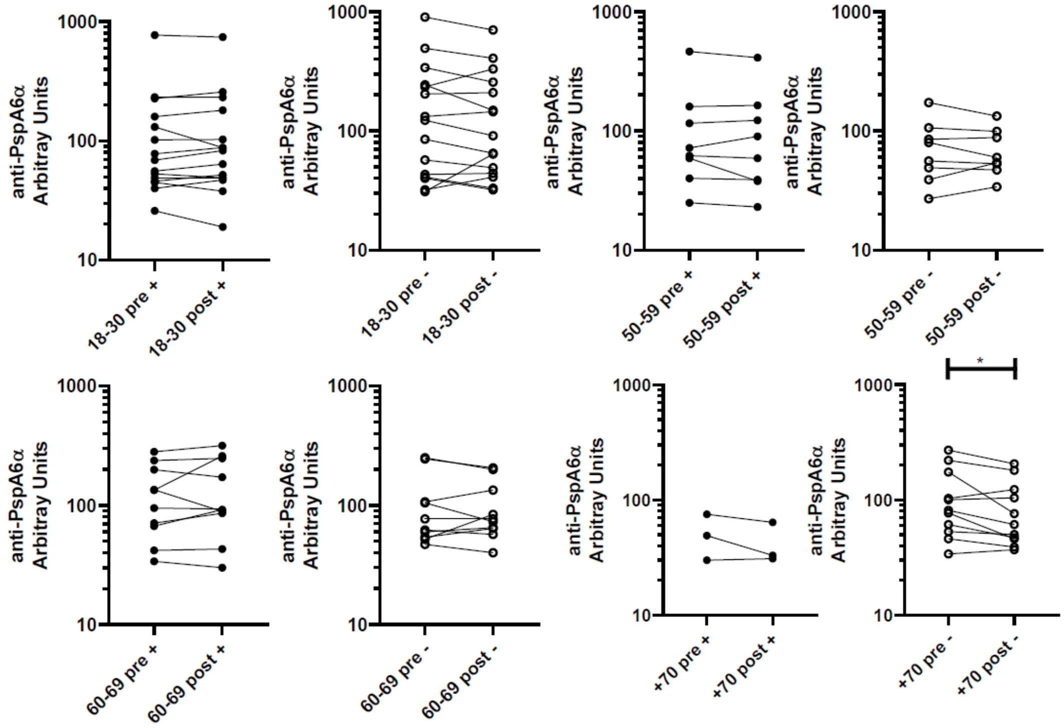

S4 Fig. Pre- and post-challenge serum levels of anti-PspA IgG in each colonization positive and colonization negative volunteer. Serum IgG against PspA1α (A), PspA2α (B), PspA3α (C), PspA4α (D), PspA5α (E) and PspA6α (F) was detected by ELISA in pre- and post-challenge serum samples of each colonization positive (+) and colonization negative (-) volunteer grouped by age. * indicates difference with statistical significance between pre- and post-challenge samples (Paired Student's *t*-test, * $P \leq 0.05$, ** $P \leq 0.01$).
