# Supplementary material for: Serum levels of anti-PspA and anti-PspC IgG decrease with age and do not correlate with susceptibility to experimental human pneumococcal colonization
Source: PLoS One. 2021 Feb 12;16(2):e0247056. doi: 10.1371/journal.pone.0247056 (PMC7880446; doi:10.1371/journal.pone.0247056)
Supplement: S5 Fig — Serum IgG against PspC3α (A), PspC6α (B), PspC8α (C) and PspC9α (D) was detected by ELISA in pre- and post-challenge serum samples of each colonization positive (+) and colonization negative (-) volunteer grouped by age. * indicates difference with statistical significance between pre- and post-challenge samples (Paired Student’s t-test, * P≤0.05, ** P≤0.01, *** P≤0.001). (PDF) [file pone.0247056.s005.pdf]

A

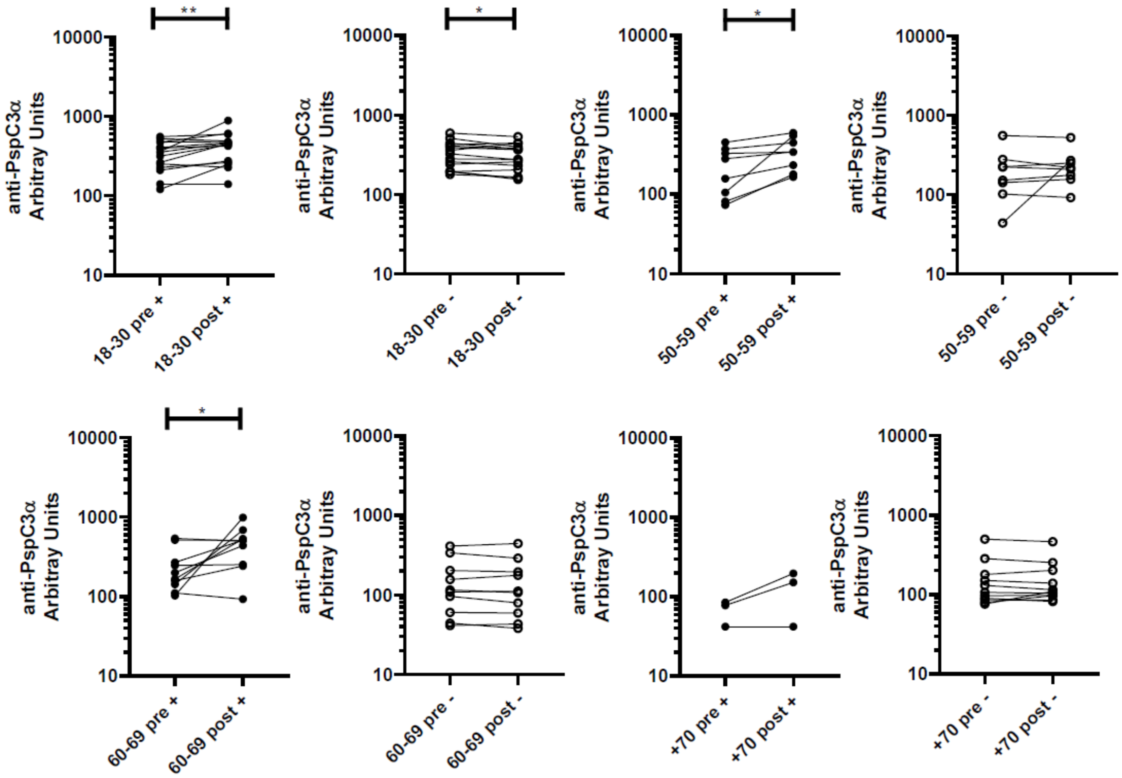

**
*
*
*
anti-PspC3α
Arbitray Units
10000
1000
100
10
18-30 pre +
18-30 post +
18-30 pre -
18-30 post -
50-59 pre +
50-59 post +
50-59 pre -
50-59 post -
60-69 pre +
60-69 post +
60-69 pre -
60-69 post -
+70 pre +
+70 post +
+70 pre -
+70 post -

B

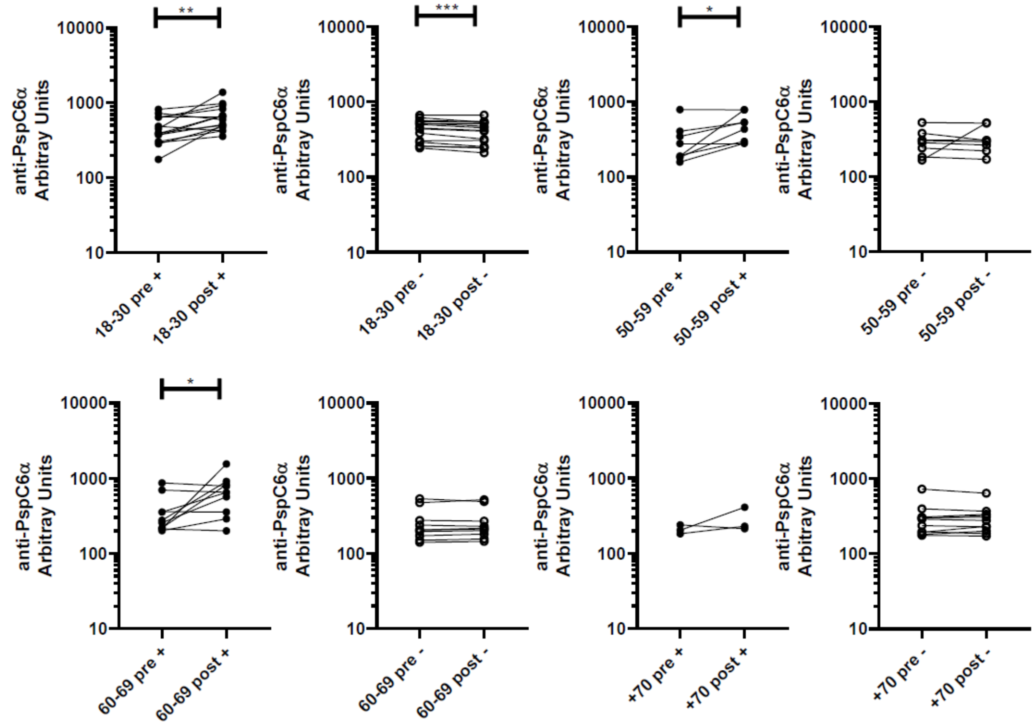

**
***
*
*
anti-PspC6α
Arbitray Units
10000
1000
100
10
18-30 pre +
18-30 post +
18-30 pre -
18-30 post -
50-59 pre +
50-59 post +
50-59 pre -
50-59 post -
60-69 pre +
60-69 post +
60-69 pre -
60-69 post -
+70 pre +
+70 post +
+70 pre -
+70 post -

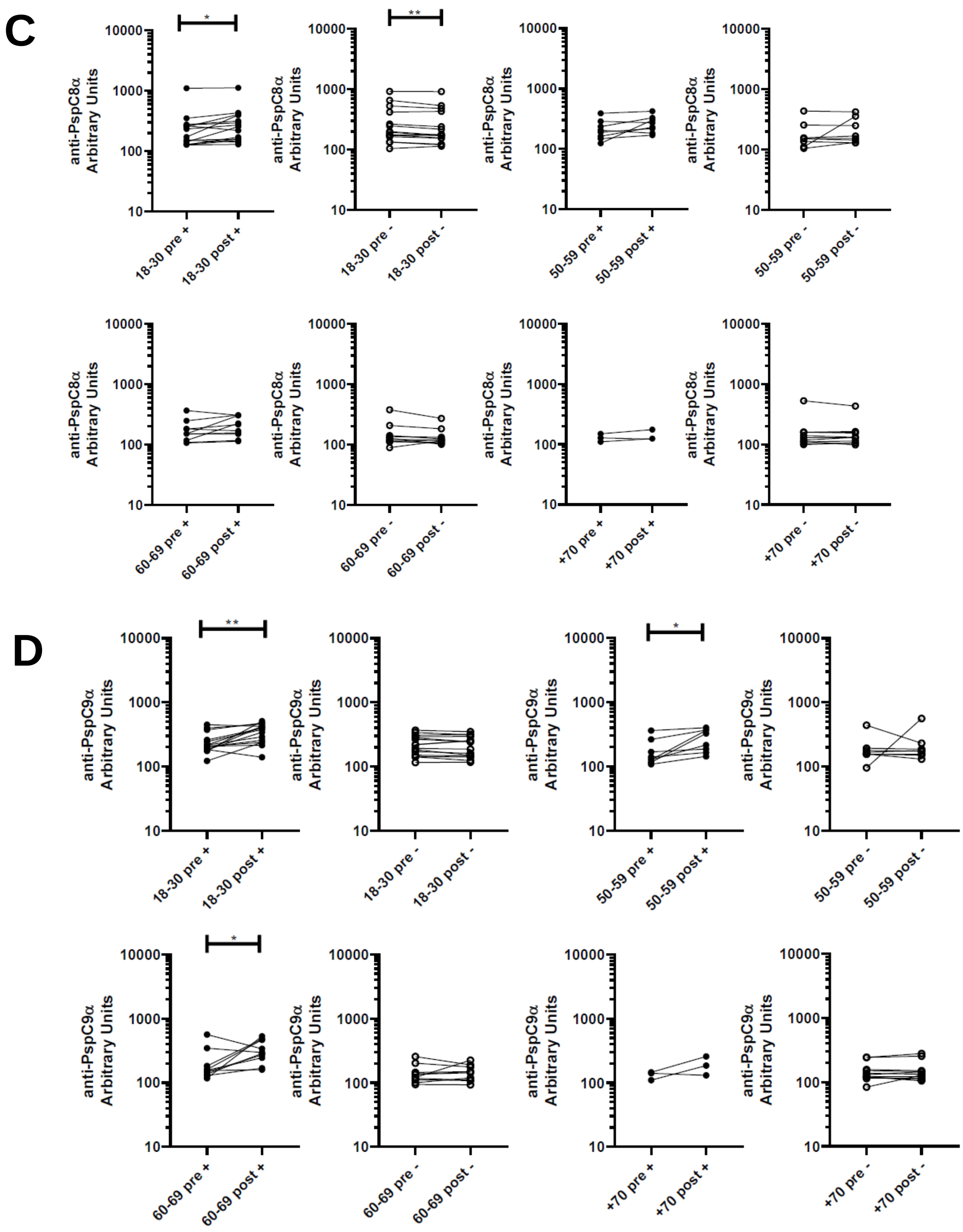

S5 Fig. Pre- and post-challenge serum levels of anti-PspC IgG in each colonization positive and colonization negative volunteer. Serum IgG against PspC3α (A), PspC6α (B), PspC8α (C) and PspC9α (D) was detected by ELISA in pre- and post-challenge serum samples of each colonization positive (+) and colonization negative (-) volunteer grouped by age. * indicates difference with statistical significance between pre- and post-challenge samples (Paired Student's *t*-test, * $P \leq 0.05$, ** $P \leq 0.01$, *** $P \leq 0.001$).
